# Dyadic Experiences and Psychosocial Management of Couples Facing Advanced Cancer: A Systematic Review of the Literature

**DOI:** 10.3389/fpsyg.2022.827947

**Published:** 2022-06-08

**Authors:** Marie Hasdenteufel, Bruno Quintard

**Affiliations:** LabPsy – Laboratoire de psychologie de l'université de Bordeaux, Unité de recherche EA 4139, Bordeaux, France

**Keywords:** advanced cancer, couple, couple's experience, couple therapy, end-of-life

## Abstract

**Background:**

Cancer diagnosis and treatment represent a real upheaval both for the patient and for his or her life partner. Adjustment to cancer has been widely studied at the individual level, however, there is little in the literature about the experiences of the couple as an entity. This is especially true with regard to a population facing advanced cancer. This systematic review aimed to make an inventory of 1) the current knowledge relating to the experience of the patient-partner dyad when confronted with advanced cancer, and 2) the psychosocial interventions specifically centered on this dyad.

**Method:**

This review was conducted using the Cochrane methodology. The eligibility criteria for the literature review were: one of the members of the dyad being treated for advanced cancer, dyad composed of the patient and his/her life partner. Databases from PubMed, PsycArticle, PsycInfo, Psychology and Behavioral Sciences Collection and Scopus were investigated. A thematic content analysis on the basis of admitted articles made it possible to respond to each of our research objectives.

**Results:**

Three hundred eighty-nine citations were found. Twenty were admitted to the systematic review of the literature. It highlighted the following experiences of the advanced cancer patient-life partner dyad: uncertainty about the future, disjointed time, intrusion into the couple's intimacy, attachment style and caregiving within the couple, couple's adjustment to cancer symptomatology, the couple's supportive care needs, role changes, nature of communication within the couple, anticipation of the coming death, and the meanings and beliefs around death. This review also describes the range of couple therapies used in the context of advanced cancer: emotionally focused-couple therapy, existential therapy, art therapy, support therapy and couple communication and intimacy promotion. These therapies seem to have individual beneficial effects for both the patient and his or her life partner as well as improving marital functioning.

**Conclusions:**

These results clearly highlight that consideration of the couple and communication within the couple during care are fundamental to dyadic adjustment to advanced cancer. Further studies (qualitative and quantitative) are needed to better understand the couple's experience in order to adapt the management of the couple facing advanced cancer.

## Background

Cancer diagnosis and treatment represent a real upheaval, both for the patient and for those around him or her (Nijboer et al., 1998; Carlson et al., 2000; Kayser et al., 2007; Mangione, 2017). It generates multiple repercussions of a psychological, physical, social and existential nature (Janda et al., 2007; Hagedoorn et al., 2008; Northouse and McCorkle, 2015). Faced with cancer and these repercussions which act as stressors, cancer patients and their family members adopt diverse individual strategies to cope (to modify the situation or to modify their reactions to make it more bearable) (Lazarus Folkman, 1984). While many studies have been carried out using this approach and focusing on the experience of the cancer patient, few studies have explored the experience of the couple, as an entity, and its adjustment to the disease (Untas et al., 2012).

Many studies showed that the wellbeing of caregivers and their loved ones with cancer are closely linked; this is particularly true when the primary caregiver is the spouse (Northouse, 1989; Baider et al., 1996; Northouse et al., 1998; Hodges et al., 2005). Both members of a couple have a mutual impact on each other's quality of life, psychological health and adaptation to their respective roles (Northouse et al., 1998; Kim et al., 2008). Cancer diagnosis and treatment then appear as stressors for the couple (Maughan et al., 2002; Dankoski and Pais, 2007).

Stress and its management would be then an interactive phenomenon between the two partners of a couple, the signs of stress of one triggering management reactions in the other (Bodenmann, 1995). Concept of dyadic coping was thus introduced and it corresponds to the set of efforts of one or both partners intended to manage stressful events, as well as the tensions experienced by one (individual stress) or by both partners (dyadic stress) (Bodenmann, 1995). It includes management strategies for maintaining or restoring the structural, functional, behavioral, emotional and social balance of the dyadic system as well as the balance of each partner (Bodenmann, 1995). If this is a first way of conceiving the dyad, we can even go further in its apprehension by taking into account the disease, the family, medical and social contexts, while specifying the relations between the patient and his/her life partner (that is to say taking into account the patient, the life partner and their relationship); each entity (patient, relative, dyad) with its own characteristics in terms of history, transactional variables and criteria (Berg and Upchurch, 2007; Untas et al., 2012). Finally, 3 concepts are fundamental to understanding the dyad (and fit perfectly into the conception presented above): “communication,” “reciprocal influence,” and “patient-caregiver congruence”^1^ (Manne and Badr, 2008; Fletcher et al., 2012; Li and Loke, 2014). Indeed, in the context of cancer, a satisfactory communication between couples is linked to less distress and better marital adjustment (Li and Loke, 2014). Multiple interactions within dyads correlate with a sense of wellbeing and dyadic adjustment (Li and Loke, 2014). And congruence in dyads is linked to better individual health-related quality of life outcomes and relationship satisfaction (Li and Loke, 2014). For example, symptoms related to cancer affect the communication and interaction within a couple (Li and Loke, 2014).

If the symptoms linked to cancer appear as predominant mediating factors in the adaptation of the dyad to cancer, they are all the more present when the patient is in the palliative phase of the disease; which may suggest that the adaptation of the couple is all the more complicated. The palliative phase of the disease can be assimilated to what is called “advanced cancer” which is defined as follows: “cancer that is unlikely to be cured or controlled with treatment; it may have spread from where it first started to nearby tissue, lymph nodes, or distant parts of the body […]” (NIH, 2011). What is more, as well as the symptomatic which worsens, the palliative phase resounds with the anticipation of the fatal outcome, which generates an intense distress for the couple (Delvaux, 2006). In addition, there are communication difficulties, difficulties relating to physical and emotional care and emotional difficulties linked, particularly in the way to feelings of separation and loss (Delvaux, 2006).

If at the individual level, we know that the palliative cancer phase exacerbates these difficulties (Weitzner et al., 1999), it is legitimate to think that at a dyadic level it is the same thing. However, there is very little evidence in this specific area of dyadic end-of-life experience. von Heymann et al. (2017) wrote that “the application of the concept of dyadic adaptation at the end of life is relatively new and the role of dyadic adaptation at very advanced stages of the disease is not clear” (von Heymann et al., 2017). It is therefore essential to take an interest in the dyadic adaptation of the patient and his/her life partner in this last phase of the life. Thus, the objectives of this systematic review of the literature were: 1) to explore the literature on the dyadic experience of the patient and his/her life partner when confronted with advanced cancer; and 2) to highlight the main psychosocial interventions offered to the patient-life partner dyad in the context of advanced cancer and what their effects are.

## Method

### Eligibility Criteria

To establish our search strategy, we relied on the PICOTS criteria (Population, Intervention, Comparison, Time, Setting) to break down the evaluation question into different concepts that we used to build the research strategy:

- Population: All studies on adult patients (>18 years old) with a diagnosis of advanced cancer (“stage III” and “stage IV” or “terminal cancer”) and their life partner (spouse, partner, husband, wife, civil union), with no limitations regarding time since diagnosis or cancer location were included. Couples could be either homosexual or heterosexual.
- Intervention: Studies relating both to (1) the couple's experience and (2) psychosocial interventions intended for the couple, in the context of advanced cancer, were included.
- Comparison: In view of the difficulty accessing the population studied, the absence of a control group was not an exclusion criterion.
- Outcomes: Studies relating to the quality of life, psychosocial aspects and symptoms were included and any studies reporting results which related to the structural, functional, behavioral, emotional and social balance of the dyadic system.
- Temporality: We did not place any time restrictions.
- Setting: The study population is accompanied by care services (ambulatory or complete) or an oncologist.

We are aware that with such broad search criteria many documents could be found (editorials, letter to the editor, open forum, news, summary articles, original articles). We wanted to make our own selection of the types of documents to integrate according to the number of results found. We made this choice with regard to the research context (end of life) in which we operate. We know that this is a context where it is difficult to conduct analytical studies.

### Source Information and Search Strategy

We queried the following electronic databases: PubMed, PsycArticle, PsycInfo, Psychology and Behavioral Sciences Collection and Scopus, with no limitation for publication date or language. The search was last updated on 31 October 2021. While the search strategies were the same for each database, they were adapted to the way the database works. The search strategy with thesaurus was as follows: “Couple” AND “palliative care” (Appendix). According to the database thesaurus, there could have been nuances in some key terms (e.g., for the expression “palliative treatment”). The search strategy without thesaurus was: “Couple” AND “palliative care” OR “end of life care” OR “terminal care” OR “dying.” Without the thesaurus, a search strategy was carried out with descriptor “Keyword” and “Subject.” All of these strategies were used in each database. This search strategy was validated by a librarian.

### Study Screening and Selection

All search results were merged into an Excel spreadsheet. Duplicates were excluded. The title and abstract of each of the articles were reviewed and those who did not meet the eligibility criteria were excluded. The process of excluding articles on the basis of title and abstract was carried out in an independent double rating, by MH and BQ on 10 references, in order to reinforce the interrater validity of this review. In case of doubt, the full texts were read. Full texts were studied for all remaining studies. Those who did not meet the eligibility criteria were excluded. The process of excluding articles on the basis of reading the full texts, was carried out in independent double rating, by MH and BQ on 10 references in order to reinforce the interrater validity of this review. For accepted articles, a characteristic table of studies was completed. We completed our search strategy by studying the bibliographies of the included studies.

Then, for each study, a distinction was made as to whether it met objective 1 (dyadic experience of the patient and his/her life partner when confronted with advanced cancer) or objective 2 (psychosocial interventions offered to the patient-life partner dyad in the context of an advanced cancer and their impact). Finally, a thematic content analysis of each study was conducted separately by two psychology researchers (BQ and MH) who then compared their results in order to identify the main themes mentioned in the literature, related to each of the two research objectives. This thematic content analysis was conducted according to the methodology proposed by Paill and Mucchielli (2021). We chose thematic content analysis because it is no longer just a question of identifying themes, but also of checking whether they are recurrent from one material to another and how they overlap, join, contradict each other, complement each other (Paill and Mucchielli, 2021).

### Critical Evaluation of Study Quality

The Critical Appraisal Toolkit was developed by a team from the Public Health Agency of Canada and a Cochrane reviewer with methodological expertise to appraise analytical studies, descriptive studies and reviews of literature (Public Health Agency of Canada, 2014). This toolkit was originally designed to evaluate evidence in the field of infection prevention and has been applied to other areas. This toolkit addresses the following points for analytic studies: participants, internal validity, confounding control, ethics, analyses, and applicability. For descriptive studies, the following points are evaluated: the participants, the sources and methods of collection, the instruments used, the ethics and the analyses. Moreover, the toolkit for descriptive analyses proposes criteria to try to understand the quality of a case report; which in our field of study seems very relevant. Finally, the Critical Appraisal Toolkit classifies the quality of studies as high (no impediment to the ability to draw a conclusion about the clear association between the exposure and the outcome under study), medium (probability that there is an association between the exposure and the outcome under study) or low (association between exposure and outcome under study is compromised). This tool therefore gives us the opportunity to assess the quality of the study but also the strength of the study design and the directness of the evidence.

This methodological quality appraisal of the included studies was performed independently by two researchers (BQ and MH). When discrepancies appeared, oral discussion of the manuscripts was performed for consensus.

## Results

### Selection and Description of Studies

**Figure 1:**
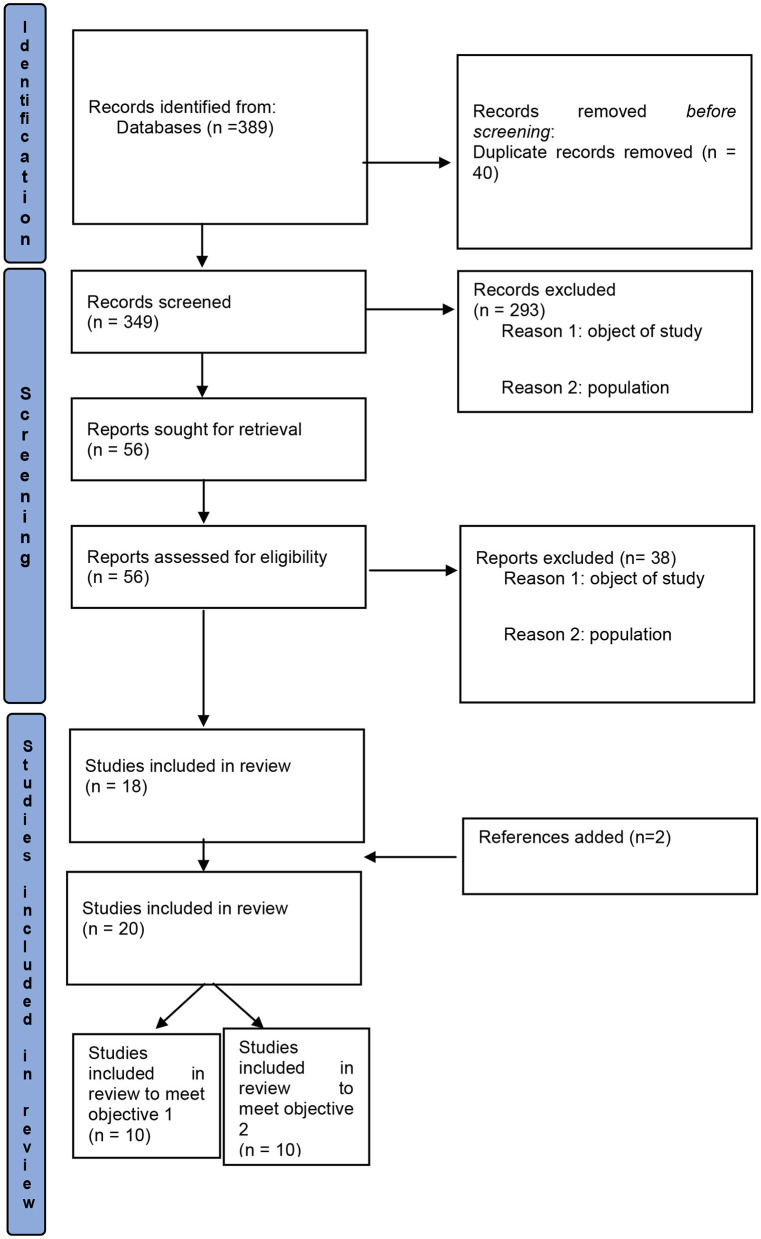
Flowchart of the study selection process.

A total of 389 citations were found through this search strategy, including 301 through PsycINFO, PsycArticle, Psychology and Behavioral Sciences Collection, 71 through PubMed and 17 through Scopus (Figure 1). After excluding duplicates (*n* = 40), 349 records were screened (titles and abstracts), which led to the exclusion of 293 studies (object of study, population). Thus, 56 full-text articles were assessed, of which 38 were excluded, leaving 18 studies. Two additional references, found in the bibliography of the articles, were included, which finally corresponded to 20 studies admitted to the review. Ten studies met our first objective and 10 studies met our second objective.

### Themes Identified in the Literature on the Dyadic Experiences of the Patient and His/Her Life Partner When Confronted With Advanced Cancer

Of the 20 studies admitted in our review, 10 met our first objective (to describe the adjustment of the patient-life partner dyad for advanced cancer):we identified 1 book chapter, 1 clinical correspondence, 1 case study, 1 review of the literature (2007), 3 qualitative studies and 3 quantitative studies (2 descriptive-cross-sectional, 1 descriptive longitudinal). Most of the studies were conducted in North America (2 in the United States, 3 in Canada). The other studies were conducted in Switzerland (*N* = 1), the United Kingdom (*N* = 1), France (*N* = 1), Belgium (*N* = 1) and Germany (*N* = 1). At least 779 couples were studied. Patients had an average age of 63.08 years. Caregivers had an average age of 60.27 years. The results of these studies are summarized in Table 1.

**Table 1:** Descriptive table of experience of patient-life partner dyad faced with advanced cancer.

**References**	**Aim**	**Population**	**Instruments**	**Data analyses**	**Results**
Opsomer et al. (2019)	To explore how couples cope with nutrition-related issues in advanced cancer	7 couples Patients: -women 57.14% -age 67.6 y -advanced cancer (multisite) Caregivers: -women 42.86% -age: NA	Semi-structured interviews (patient and his or her partner were interviewed concurrently)	Qualitative Analysis Guide of Leuven (QUAGOL)	Overarching coping strategies: to maintain routines and normality in daily life; to create new routines or a new normality. Disclosure of nutrition-related problems: overt communication, presenting the problem the way it appears; overt communication of problem together with its solution or how to deal with it; hiding problem from the partner and disclosing it during the interview; exposing problem because it is too obvious to hide. Couple-coping pathways: practical oriented (action of partner: adapting food; taking over daily tasks; searching for a practical solution; making it easy for the patient) (reaction of the patient: accepting help; not accepting help); emotion oriented (action of partner: emphasizing the severity, confirming empathically, insisting) (reaction of the patient: confirming the words of the partner, toning down the words of partner, contradicting the word of partner); distance oriented (action of partner: withdrawing, not responding) (reaction of partner: coping individually)
Gardner (2008)	To explore patterns of relationships, support and communication in married couples or couples where one partner is diagnosed with advanced and terminal cancer	35 couples Patient: -women 29% -age 66 y -advanced cancer (multisite) Caregiver: -women 71.43% -age 65 y	Semi-structured interviews (each patient and partner caregiver met face-to-face with an interviewer, first together and then for separate interviews)	Grounded theory analytic methods	Individual and dyadic processes: existence of individual and dyadic discourses, where patient and partner respondents moved fluidly and repeatedly between individual and dyadic frames of reference. Living with uncertainty: most common concerns that patients and caregivers described was the struggle to deal with the uncertainty and ambiguity surrounding the patient's medical condition and future. Illness and dying trajectories: awareness of death pervaded the responses of study participants and its interrelated themes of uncertainty about the future. Search for shared meanings: despite differences in personal awareness and acceptance about death, some couples seemed to be working toward a shared understanding, narrative, or philosophical approach related to the patient's illness trajectory and ultimate prognosis.
Weißflog et al. (2017)	To evaluate the levels of dyadic coping and supportive care needs and their concurrent associations	330 couples Patients: -women 36.7% -age 57 y -advanced cancer (hematologic) Caregiver: -women 63% -age 56 y	Dyadic coping inventory (DCI) Supportive care needs survey-short form German version (SCNS-SF-34-G)	Actor-partner interdependence models (APIM)	Perception of partners' delegated dyadic coping was higher. Higher perceptions of partners' negative dyadic coping were associated with higher supportive care needs for both patients and partners. Higher perceptions of patients' own stress communication and supportive care need, but only for the patients.
Mah et al. (2020)	To evaluate a moderated mediation model in which perceived couple communication mediates the relationship between attachment security and death preparation in individuals with advanced cancer and in which gender and age moderate these relationships.	Participants -women 55.4% -age 58.10 y	Quality of Life at the End of Life Cancer Scale (QUAL-EC) Experiences in Close Relationships Scale (ECR). Couple Communication Scale Patient Health Questionnaire (longitudinal data: baseline, 3 and 6 months)	Mediation and moderated mediation	Couple communication mediated the relationship of attachment security to preparation for end of life and life completion Anxiety and Gender effects on baseline couple communication: indicated that women with greater attachment anxiety reported worse communication than their male counterparts. Couple-communication, gender and age effects on baseline preparation for end of life: suggested that women showed better preparation with better couple communication. Younger patients reported less preparation than older patients, especially with poorer communication, but their preparation increased with better communication, especially in younger men.
Braun et al. (2012)	To examine associations between caregiving styles and caregivers' and patients' attachment orientations among couples facing advanced cancer.	110 couples Patients: -women NA -age 61.7y Advanced cancer (lung cancer gastrointestinal) Caregivers: -women 76.9% -age 59.8 y	Experiences in Close Relationships inventory (ECR) Caregiving Questionnaire Demand Subscale from the Caregiving Burden Scale	Hierarchical regressions	Caregivers reported high levels of proximate and sensitive caregiving and moderate levels of controlling and compulsive caregiving. Both caregiving proximity and sensitive caregiving were negatively associated with caregivers' avoidant attachment. Controlling caregiving was positively related to caregivers' avoidant and anxious attachment orientations. Compulsive caregiving was positively associated with caregiving demand and caregivers' attachment anxiety. Compulsive caregiving was positively associated with patients' attachment avoidance and negatively associated with patients' attachment anxiety.
Reny (2020a)	To examine how the couple is accompanied when faced with the end of life	Patient: -women: 25% -age: NA -advanced cancer (multisite) Caregiver: -women: 75% -age: NA	Case study		Time-sharing no longer took the same signification. Achievements such as complicity, intimacy, being together were called into question. Complete fusion of couple VS phenomenon of distancing. The roles of each become different. Patients and spouses report actively hiding negative emotions and grief from their counterparts to avoid worry about each other. Talking to each other, understanding each other becomes more complex. The disease breaks a part of illusion: soon the couple will no longer be.
McLean and Jones (2007)	To provide an overview of the impact of cancer on the couple,	End of life cancer	Review		Major depressive syndromes, anxiety, and role adjustment problems: patients and their spouses (increases as death approaches). Similarities in terms of distress response between patients and their spouses. Factors could explain distress: patient's condition (demographic, and psychological factors, social support and resources), level of marital satisfaction, quality of family functioning, difficulties in the ease of couples communicating cancer-related concerns, high conflict, low expressiveness, low cohesion (and the other hand: high levels of support, cohesion, expressiveness, and low conflict, positive emotional environment could help). Secure marital bond: attachment insecurities and behaviors are adequately addressed within the relationship (and the other hand: with insecure marital bond, attachment insecurities and behaviors may be expressed in maladaptive patterns of interaction that maintain separation distress). Attachment and caregiving styles are closely linked: secure attachment is linked to highly responsive care while, insecure attachment is linked to a low level of responsive care. Unresolved issues (with emotions, such as anger, sadness and longing, shame, and fear) in the marital relationship can either pose as a significant threat to the attachment bond, or an opportunity for further growth and development. Patients facing end of life express concerns regarding their spouses and families + the desire to strengthen relationships (these concerns can often exceed disease-related concerns). Patients and spouses may seek increased avoidance, or proximity and closeness to each other.
Drabe et al. (2016)	To gain a deeper understanding about couples' relationship changes over time after one partner is diagnosed with an incurable advanced melanoma.	8 couples	Semi structured interviews (longitudinal data: baseline, 6 months)	Qualitative content analysis	Baseline: relationship changes reported in terms of caring, closeness/distance regulation, and communication patterns. 6 months: relationship changes reported in terms of caring, distance/closeness regulation, greater appreciation of the relationship and limitations in terms of planning. 50% of patients and partners: hiding their negative emotions and sorrows from their counterparts to spare them worry.
Iwasaki et al. (2018)	To discuss the existential questions of patients and their partner facing the end of life	Advanced cancer	Clinical correspondence		Patients are concerned about the future of their family members, especially their spouses. End-of-life discussions often remain practical in nature (pain relief, funeral arrangements, distribution of belongings, etc.). Scaffolding for communication about ”what about you after I'm gone¿‘ can be important. Discussing a spouse's future intimate relationships and happiness could ease deep concern and existential distress in dying patients; it helps the surviving partner to feel less distressed if such opportunity arises Having the agreement or permission of a dying patient may reduce the possible negative consequences associated with a new romantic relationship if it is continued. Such a conversation may reduce existential distress, increase peace of mind and bring the dying patient to a state of relief.
Cort et al. (2004)	To describe the sexual and intimacy needs of the couple when one partner has a terminal illness	Advanced cancer	Chapter of book		The myth of cancer contagiousness and fear of pain can drive the couple away from sexuality. Sexual problems often arise from interpersonal problems to which both partners contribute. Diagnosis of cancer could allow couples to re-examine their relationship and move forward in a positive way (reverse is also true).

The results of the quality assessment are summarized in Table 2. Very high inter-rater agreement was obtained. Two studies were “high quality.” Four studies were “moderate quality” due to missing information concerning ethics, tools whose validity and reliability have not been demonstrated but whose validity can be believed in the light of the questions asked and the expertise of researchers, analyses that cannot demonstrate the effect with certainty, or non-random sampling. Finally, four studies (1 case study and 3 reviews) were characterized as having “low quality”: the reviews because they were narrative reviews; the case report because the quality (particularly at the level of the analysis) was not high. While for six of these studies, the quality is “good” to “moderate,” the research plan remains is “low” because of the design of the study (descriptive study).

**Table 2:** Quality assessment of the included studies of dyadic experiences of the patient and his/her life partner when confronted with advanced cancer.

**References**	**Type of studies**	**Research question**	**Particip-ants**	**Sources and methods**	**Tools**	**Ethics**	**Analyses**	**Study plan strength**	**Quality of study**	**Directness of evidence**
Opsomer et al. (2019)	Descriptive	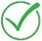	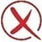	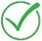	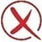	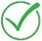	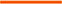	Low	Moderate	Direct
Gardner (2008)	Descriptive	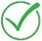	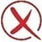	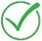	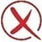	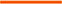	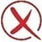	Low	Moderate	Direct
Weißflog et al. (2017)	Descriptive	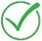	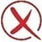	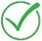	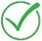	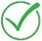	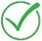	Low	High	Direct
Mah et al. (2020)	Descriptive	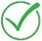	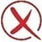	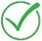	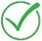	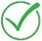	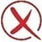	Low	Moderate	Direct
Braun et al. (2012)	Descriptive	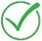	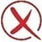	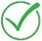	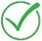	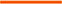	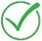	Low	High	Direct
Drabe et al. (2016)	Descriptive	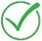	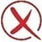	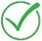	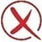	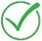	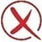	Low	Moderate	Direct
**References**	**Type of studies**	**Participants (case report)**	**Quality (case report)**					**Conclusion**		
Reny (2020a)	Descriptive (case report)	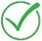	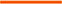					This case study suggests lines of thought relating to the phenomenon under study. It is necessary to carry out more robust studies in order to have a sufficient level of proof to validate the hypotheses put forward.		
**References**	**Type of studies**	**Research question**	**Included Studies and Critical Appraisal**					**Conclusion**		
Iwasaki et al. (2018)	Review	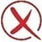	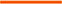					This review suggests lines of thought relating to the phenomenon under study. It is necessary to carry out more robust studies in order to have a sufficient level of proof to validate the hypotheses put forward.		
Cort et al. (2004)	Review	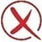	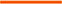							
McLean and Jones (2007)	Review	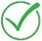	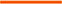							

*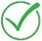: strong/high*

*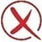: moderate/medium*

*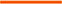: weak/low*

Couples in which one partner had been diagnosed with advanced and terminal cancer report both their individual (intra-personal) and shared (dyadic) experiences (Gardner, 2008). While most of the time the discourses of patients and their spouses agreed, there may be some discrepancies (e.g., death, beliefs, etc.) (Gardner, 2008). The systematic analysis of the issues faced by these couples could be grouped into ten main themes which are summarized as follows: uncertainty about the future, disjointed time, intrusion into the couple's intimacy, attachment style and caregiving within the couple, couple's adjustment to cancer symptomatology, the couple's supportive care needs, role changes within the couple, nature of communication in the couple, anticipation of the coming death, and the meanings and beliefs around death.

#### Uncertainty About the Future

One of the most common concerns described by patients and spouses was the struggle to deal with the uncertainty and ambiguity surrounding the patient's health status and future (and thus the partner's own future) (Gardner, 2008; Weißflog et al., 2017). This concern could weigh on the relationship (Gardner, 2008).

#### A Disjointed Time

Faced with serious illness, time-sharing no longer took the same signification (Reny, 2020a). The patient and their partner found themselves in a time that could no longer be joined (Reny, 2020a). The crisis caused by the disease generated a feeling of rupture (Reny, 2020a). Nothing was the same as before (Reny, 2020a).

#### Intrusion Into the Couple's Intimacy

Changes in relationships were related, among other things, to treatment (Drabe et al., 2016). Indeed, illness and treatment intruded into the realm of the couple just as they burst into the body of the patient (Reny, 2020a). Complicity, intimacy and being together were harder to achieve (Reny, 2020a). Intimacy is the deepest thing in ourselves, the most secret, it is above all what we do not share, or only if we decide, with those who we choose (Reny, 2020a). The intimate refers to the hidden, to the personal, to what cannot be seen in the eyes of all (Reny, 2020a). Therefore, the intimate is not reduced to the sexual (Reny, 2020a). Both intimacy and sexuality, are damaged by illness and care. The myth of cancer contagion and fear of pain can drive the couple away from sexuality (Cort et al., 2004). Irrespective of whether the cancer site involved the sexual organs, sexual self-esteem and functioning can be impaired (Cort et al., 2004). Sexual problems often arise from interpersonal problems to which both partners contribute (Cort et al., 2004). In some cases, the diagnosis of cancer could allow couples to re-examine their relationship and move forward in a positive way (Cort et al., 2004). The reverse is also true (Cort et al., 2004).

Depending on the functioning of the couple, the defenses against the intrusion of the disease and treatment, can be more or less extreme (Reny, 2020a). Some couples will need to fight against the disease by being in complete fusion, to “become one” in the face of the intrusion of the disease and the treatment associated with it (Reny, 2020a). While others will, on the contrary, be in a distancing mode to protect themselves from too much anxiety (Reny, 2020a). The regulation of proximity/distance within the couple appears to be a fundamental issue for the couple (Drabe et al., 2016).

#### Attachment Style and Caregiving Within the Couple

The regulation of proximity/distance can be explained, among other things, by the type of attachment between partners. Several studies have worked on the type of caregiving^2^ according to attachment style (McLean and Jones, 2007; Braun et al., 2012). Research has shown that attachment and caregiving styles are closely related and predictive of marital satisfaction. Avoidant attachment of spouses was negatively correlated with proximal and sensitive care (Braun et al., 2012). Anxious attachment of spouses and demand for care were positively associated with compulsive care (Braun et al., 2012). Avoidant and anxious attachment of spouses was positively correlated with controlling care (Braun et al., 2012). Finally, compulsive care provided by the caregiver was positively associated with avoidant attachment of patients and negatively associated with anxious attachment of patients (Braun et al., 2012). Another study showed that secure attachment was correlated with highly reactive care (a composite of proximity, sensitivity, and cooperation) and insecure attachment with reactive care (McLean and Jones, 2007).

#### Couple Adjustment to Cancer-Related Nutrition Issues

Daily life as a couple was also seriously threatened by nutrition-related problems and only one study addresses this issue. Opsomer et al. (2019) report that in their attempt to cope with nutrition issues threatening their health, couples seem to adopt three different couple coping paths: practice-oriented, emotionally-oriented, or distance-oriented. Each consists of an action of the partner followed by a reaction of the patient. The practice-oriented path is characterized by the partner trying to offer practical help (e.g., by adapting the patient's diet or taking care of daily tasks), followed by the reaction of the patient who has often accepted the proposed help. In the emotion-driven journey, the partner's action is communicative, emphasizing the severity of symptoms, making empathetic responses, or insisting that the patient eats. Such a communicative action is usually followed by a communicative reaction on the part of the patient: it confirms the partner's words, attenuates them or contradicts them. For the distance-oriented pathway, the partner withdraws or does not take any action. Therefore, the patient must cope alone.

#### Couple Adjustment and Supportive Care Needs

Levels of dyadic adaptation appear to be related to supportive care needs (Weißflog et al., 2017). High perceptions of partners' negative dyadic coping were associated with high support care need for both patients and partners (Weißflog et al., 2017). The same was true for patients' own stress communication and support care need, but only for the patients (Weißflog et al., 2017).

#### Roles Changes Within the Couple

Treatment and illness generate an asymmetry between the ill partner and the one who is (assumed to be) healthy (Reny, 2020a). Faced with illness, couple dynamics change; roles change and the weight of guilt, even debt can be experienced (Cort et al., 2004; Reny, 2020a). While communication (e.g., open, empathetic, on existential questions, on fears and the changing perception of time, on the concrete modalities of the end of life, but also reflecting back on married life) within the couple promotes adaptation to changes in roles, it also appears to be a key element in the adoption of more adaptive coping strategies, and in the satisfaction and the quality of the conjugal relationship (McLean and Jones, 2007).

#### Communication Within the Couple

Communication is central to the intimate relationships. Following the diagnosis of advanced cancer, communicational patterns within the couple change (Drabe et al., 2016). Patients and spouses report actively hiding negative emotions and grief from their counterparts to avoid worrying each other (Cort et al., 2004; Drabe et al., 2016; Reny, 2020a). Talking to each other, understanding each other becomes more complex (Reny, 2020a). Difficulty communicating about cancer problems can lead to emotional insecurity, distress and relationship instability (McLean and Jones, 2007). Conversely, a good level of communication contributes to the proper functioning of the couple (as well as a high level of support and cohesion and less conflict), which will in turn reduce the level of distress, anxiety and depression (McLean and Jones, 2007).

It turns out that end-of-life communication is essentially focused on the practical dimension (e.g., pain relief, funeral arrangements, distribution of personal belongings, etc.) (Iwasaki et al., 2018). However, one study demonstrates that discussion between patient and spouse about the surviving spouse's romantic future would help reduce the negative consequences associated with a new romantic relationship in which the surviving spouse may become involved (Iwasaki et al., 2018). Such a conversation can reduce existential distress, increase peace of mind, and bring relief to the dying patient (Iwasaki et al., 2018). Finally, another study highlights that open couple communication mediates the relationship between attachment security and end-of-life preparation (Mah et al., 2020). Specifically, the interaction between anxious attachment and gender influences communication within the couple: women with anxious attachment reported worse dyadic communication than their male counterparts. In addition, Mah et al. (2020) showed that couple communication, gender and age influence preparation for the end of life. Partners of women showed better preparation than partners of men for the end of life (in connection with better couple communication). In the same study, younger patients reported less end-of-life preparation than older patients (related to poor couple communication), but their end-of-life preparation increases with better couple communication, especially in younger men.

#### Anticipation of Upcoming Death

Couples are not prepared to anticipate the death of the other (Reny, 2020a). The couple is defined as the union of two people by means of a “love bond marked by an avowed or undeclared intention to last” (Reny, 2020a). The possibility of the death of the other impacts this primary intention of the couple (Reny, 2020a). The disease breaks a part of the illusion: soon the couple will no longer be (Reny, 2020a). It is a moment of doubt about the value that the other can bring us and about the value we can also give (Reny, 2020a). While death consciousness is a central concern for the patient and his/her spouse, it can be denied and distanced from couple discussions (Gardner, 2008). For many patients, the subject of death is closely linked to concrete concerns about the wellbeing of their partners, children and grandchildren (Gardner, 2008). For caregivers, the problem of the future without the loved one is essential (Gardner, 2008). Despite differences in personal awareness and acceptance of death, some couples are working toward a common understanding, narrative or philosophical approach of the future, related to the trajectory of the patient's disease and ultimate prognosis (Gardner, 2008). A new layout is necessary, a new way of being together is worked (Reny, 2020a).

#### The Meanings and Beliefs Around Death

While spouses share personal and shared beliefs about health and disease in relation to cancer, the most common is the importance of maintaining a positive or optimistic attitude (Gardner, 2008). Many considered positive thinking as a method of control in the face of an uncertain course and prognosis of the disease (Gardner, 2008). Patients and spouses talked about working together to maintain a positive approach and value mutual optimism (Gardner, 2008). Many participants relied on faith to make sense of their situation (Gardner, 2008). For couples with differing beliefs, lack of shared meaning sometimes interfered with mutual support (Gardner, 2008).

To summarize, advanced cancer and the care it requires have an impact on both the individual and the couple. As reported in the selected studies in the field, the couple facing death is confronted with a multitude of questions and changes related to the future, time, intimacy, roles, nutrition, confrontation with death and communication. Communication, supportive care needs, the need for dyadic optimism, the type of caregiving are all ways in which the couple deals with these issues and changes. For these reasons offering couple therapy in this difficult time can lead to a reduction in psychosocial distress and may actually offer an opportunity for relational growth during the later stage of life (Murillo and Holland, 2004; Hodges et al., 2005). We will discuss types of interventions in the following section.

### Impact of Dyadic Psychosocial Interventions on the Couple's Experience of Advanced Cancer

Of the 20 studies included in this review, 10 met our second objective (to describe couple-centered interventions dealing with advanced cancer): 5 case studies, 2 qualitative studies, 3 quantitative studies (2 descriptive-cross-sectional and 1 experimental with a randomized controlled trial). Most studies were conducted in America (3 = USA, 4 = Canada). The other studies were conducted in the United Kingdom (*N* = 1), Switzerland (*N* = 1) and France (*N* = 1). At least 69 couples were studied. Patients had an average age of 61.28 years. Caregivers had an average age of 55.59 years. We detailed the methodology and results of each of the included studies in a comparative table (Table 3). Five categories based on the type of intervention emerged: emotionally focused-couple therapy, existential therapy, art therapy, support therapy, and couples' communication and intimacy promotion. They are presented below.

**Table 3:** Descriptive table of interventions focused on the patient-life partner dyad faced with advanced cancer.

**References**	**Disease**	**Intervention**	**Population**	**Instruments**	**Data analyses**	**Significant results**	**Non-significant results**
Mohr et al. (2003)	Metastatic cancer	8 sessions of 50/60 min. 1/week. Reduction of distress in the couple, improving communication, and increasing intimacy to the degree that these are goals of the couple. Facilitate change of meaning (beliefs, goals, values). Increase intimacy, emotional support, reciprocity. Facilitate discussion of death and dying. Facilitate discussion about children.	6 couples Patients: -women 66.7% -age 49.3 y Caregivers: -women 33.3% -age 50.1 y	Death anxiety and worrying Depression: Beck Depression Inventory-II (BDI-II) Quality of life: global QOL Relationship quality: Positive relationship and negative relationship Social support: Perceived Spousal Support Scale (positive support and negative support). Caregiver burden: Zarit Caregiver Burden	Effect sizes	Patients: Decreased distress about dying Improved positive relationship Partners: Decreased frequency of worry about dying	Patients: Worry about dying Depression Quality of life Relationship Negative Positive support Negative support Partners: Distress about dying Depression Quality of life Caregiver Burden Relationship positive Relationship Negative Positive support Negative support
McWilliams (2004)	Terminal breast cancer	Psychotherapy based in attachment theory aimed at increasing intimacy	1 couple: 1 female patient aged 83 y, male caregiver aged 81 y	Case study		Psychological growth and preparation for future bereavement. To trust each other more and to trust that they could continue to grow as a couple even though their time was limited.	
Mowll et al. (2015)	Advanced cancer	PDI–CI intervention (to improve communication around end-of-life issues for couples where one has advanced cancer) 1 session of 1 h.	9 couples: Patients: -women 55% -age 64 y Caregivers: -women 44.4% -age 64 y	Semi-structured interviews	Thematic analysis	The intervention allowed the men to speak. Men and women in couples expressed that the structure of the PDI-CI is particularly useful for men to discuss issues. The intervention helped lift the veil on the feelings of each other. A number of couples reported that the intervention highlighted areas of difference between them, which then made it easier to clarify communication at that time or afterwards. The intervention facilitated changes in behavior toward others. A couple said that discussing the PDI-CI questions made the patient recognize her deteriorating health and accept more help from her husband. A patient from another couple noticed changes in the way her husband looked after her. The importance of the intervention to help prepare for the end of life was also emphasized. The intervention validated an already functional mode of communication. A number of couples felt that the intervention improved their already good communications, which aroused positive feelings. Through the intervention, more than half of the participating couples expressed that they could return to see the psychologist.	
Benzein and Saveman (2008)	Multisite cancer	Conversations about hope and suffering 3 sessions every 2 weeks	6 couples Patients: -women 83.3% -age 52–84 years	Semi-structured interviews	Thematic analysis	Couples feel that they were part of a trustful relationship, and that it was a healing experience. Opportunity to unburden themselves. Way of learning and finding new strategies for managing daily life.	
Lantz and Ahern (1998)	Advanced cancer	Existential psychotherapy (re-collection)		Case study		To reduce the meaninglessness and the symptoms and problems around meaningless that often develop around the time of the death of a family member. To help the couple facing death to remember, find, discover, confirm, and honor meanings that have been reaffirmed and deposited in the eternity of the past.	
Wagner et al. (2016)	Various forms of cancer	Existential psychotherapy: to increase meaning in life and sense of transcendence, determine wishes and hopes, and help patients and their partners communicate more openly about death and dying. 4 sessions of 60 min.	12 couples Patients: -women 63.4% -age 59.1 y Caregivers: -women 54.4% -age 59.6 y	Anxiety and Depression: the Hospital Anxiety and Depression Scale (HADS) Meaning: Meaning/Peace subscale of the Functional Assessment of Chronic Illness Therapy Spiritual Well-Being scale (FACIT-Sp) Appraisals: Cognitive Appraisals of Health scale (CAHS) Transcendence: The Missoula Vitas Quality of Life Index (M-VITAS) Interview (assess satisfaction of intervention)	Descriptive statistics and paired samples *t*-tests. Thematic analysis.	Partners: Decreased anxiety and depression Secondary Appraisals;Increased peace with Illness Patients: Decreased threat appraisals	Partners: Meaning/Peace Threat appraisals Harm/Loss Appraisals Challenge Appraisals Patients: Anxiety and depression Meaning/Peace Harm/Loss Appraisals Challenge Appraisals Secondary Appraisals Peace with Illness Transcendence
Reny (2020b)		Support		Case study		Allows ”emotional discharge.“ Means of recirculating the word within the couple. Allows (through the support and mediation offered) that the privacy of the subject and the couple is heard and recognized in the face of the invasion of hospital and caregivers within the couple. Opportunity to mourn the couple before the illness. A new arrangement is necessary, a new way of being together is being worked on. Preventively: promotes transmission and support for future bereavement for the loved one.	
McLean and Nissim (2007)	Metastatic ovarian cancer	Emotionally focused couple therapy (modified for the advanced cancer population). To facilitate marital relationships by changing habitual and distressing patterns of interaction, to increase mutual understanding and emotional engagement, and to strengthen the marital bond. 8 sessions.	Patient: -women:100% -age: 60 Caregiver: -women: 0% -age:30 years	Case study		Breakthrough in their distress pattern and an internal shift in consciousness that allowed them to respond more effectively, sharing more primary feelings than secondary defensive reactions. They both experienced a new sense of control in their ability to defuse a painful cycle. More support, empathy and love evident in their interactions. Need for multidisciplinary support was more than necessary in view of the increasingly important physical symptoms over time generating intense distress	
McLean et al. (2013)	Metastatic cancer	Emotionally focused couple therapy (modified for the advanced cancer population). To facilitate marital relationships by changing habitual and distressing patterns of interaction, to increase mutual understanding and emotional engagement, and to strengthen the marital bond. 8 sessions of 60 min. 1/week.	42 couples (22 Intervention Group; 20 Control Group) Patient IG: -women:29% -age:51.83 y Caregivers IG: -women: 24% -age: 48.82 y Patient CGs: -women: 26% -age: 49.45 y Caregiver CGs: -women: 21% -age:50.89	Marital functioning: Revised Dyadic Adjustment Scale Depression: Beck Depression Inventory-II (BDI-II) Hopelessness: Beck Hopelessness Scale (BHS) Empathic caregiving (patient): Relationship-Focused Coping Scale(RFCS) Caregiver burden (caregiver): Two subscales (Demand/Difficulty) of the Caregiver Burden Scale	Descriptive and inferential statistics (ANCOVAs)	Improved marital functioning Improved patient's perspective of caregiver‘s empathic behavior	Depression Hopelessness Caregiver burden time Caregiver burden difficulty
Clements-Cortes (2011)	Multisite cancer	Music therapy	2 couples Patient -women: 50% -age: 77 y Caregivers –women: 50%	Semi-structured interviews were conducted with participants and coparticipants	Thematic analysis	The results indicate that examining life, signing songs and creating musical gifts were central to each participant's process. Love was the central feeling that had to be conveyed by all participants to help them complete their relationships. Grief were part of the experiences of all participants. The sub-themes of strength/hope, denial, fear/pain, and knowledge can be linked to it. People who are going through their last weeks and days often express intense gratitude for their lives and for the people they have known. Each participant grew in their understanding of the importance of engaging in the completion of the relationship with the key people in their life. All of the participants also used their last weeks and days to live instead of waiting to die. They were open to growth, learning and the possibility of transformation. Strength / hope animates couples facing the end of life, just like courage and strength. Inherent in accepting your diagnosis of terminal cancer is the awareness to say goodbye to family and friends, and ultimately to life as the person knows it. Although it was difficult for the participants to say goodbye verbally, their actions show that they were doing just that.	

The results of the quality assessment are summarized in Table 4. Very high inter-rater agreement was obtained. Two studies were characterized as “high quality”(of which one case report that used the most robust methodology possible). Four studies were defined as having a “moderate quality” with regard to a multitude of criteria (ethics, tools, analyzes, power and effect size, comparability, information bias, etc.). Finally, four studies (case reports) were characterized as having a “low quality” because the quality, particularly at the level of the analysis, was not high. Although for 6 of these studies, the quality is ”high“ to ”moderate,“ the research plan remains is predominantly “low” because of the design of the study (non-comparative before-and-after study, descriptive study). Only 1 study was a randomized controlled trial.

**Table 4:** Quality assessment of the included studies of dyadic interventions of the patient and his/her life partner when confronted with advanced cancer.

**References**	**Type of studies**	**Research question**	**Participants**	**Sources and methods**	**Tools**	**Ethics**	**Analyses**	**Participants (case report)**	**Quality (case report)**							**Study plan strength**	**Quality of study**	**Directness of evidence**
Mowll et al. (2015)	Descriptive	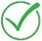	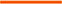	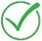	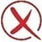	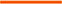	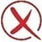									Low	Moderate	Direct
Benzein and Saveman (2008)	Descriptive	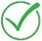	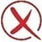	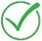	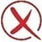	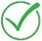	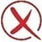									Low	Moderate	Direct
**References**	**Type of studies**	**Participants (case report)**	**Quality (case report)**													**Conclusion**		
Lantz and Ahern (1998) McWilliams (2004) Reny (2020b) McLean and Nissim (2007)	Descriptive (case report) Descriptive (case report) Descriptive (case report) Descriptive (case report)	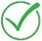 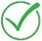 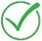 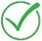	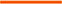 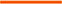 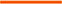 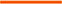													This case report suggests lines of thought relating to the phenomenon under study. It is necessary to carry out more robust studies in order to have a sufficient level of proof to validate the hypotheses put forward.		
Clements-Cortes (2011)	Descriptive (case report)	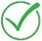	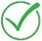															
**References**	**Type of studies**	**Research question**	**Participants**	**Selection bias**	**Misclassification bias**	**Information bias**	**Tools**	**Storage and monitoring**	**Comparability**	**Confounding variables**	**Ethics**	**Analyses**	**Power and size effect**	**Generalization**	**Feasibility**	**Study plan strength**	**Quality of study**	**Directness of evidence**
Mohr et al. (2003)	Analytique (ENCAA)	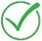	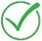	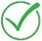	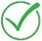	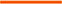	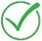	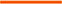	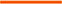	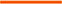	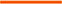	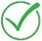	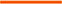	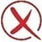	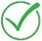	Low	Moderate	Direct
Wagner et al. (2016)	Analytique (ENCAA)	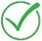	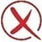	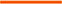	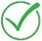	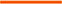	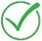	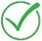	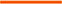	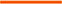	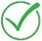	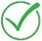	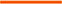	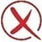	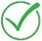	Low	Moderate	Direct
McLean et al. (2013)	Analytique (ECR)	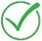	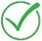	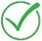	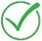	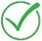	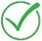	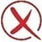	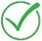	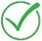	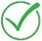	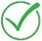	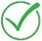	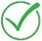	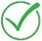	High	High	Direct

*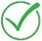: strong/high*

*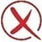: moderate/medium*

*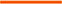: weak/low*

#### Emotionally Focused-Couple Therapy

Two studies used emotionally focused-couple therapy (modified for advanced cancer population) (McLean and Hales, 2010; McLean et al., 2013). This therapy aims to facilitate marital relationships by changing habitual and distressing patterns of interaction, to increase mutual understanding and emotional engagement, and to strengthen the marital bond. Couples benefited from 8 sessions. There was an improvement in marital functioning and the patients perceived their partners' behavior as more empathetic.

#### Existential Therapy

Two studies explored existential therapy (Lantz and Ahern, 1998; Wagner et al., 2016). This therapy aims to increase meaning in life and sense of transcendence, determine wishes and hopes, and help patients and their partners communicate more openly about death and dying. With the intervention, the loss of meaning and the issues generating the loss of meaning were reduced (Lantz and Ahern, 1998). In addition, there was a decrease in anxiety and depression among caregivers, and an increase in feelings of peace about the illness and perceptions of coping ability (secondary assessments) (Wagner et al., 2006). For patients, threat assessment decreased (Wagner et al., 2006). A third study used an intervention echoing existential therapy as it related to an intervention with conversations centered on hope and suffering (Benzein and Saveman, 2008). With this intervention couples felt that they engaged themselves in a trustful relationship, and that it was a healing experience. They had opportunity to unburden themselves. It was a way of learning and finding new strategies for managing daily life.

#### Art Therapy

Only one intervention involving art (in the broad sense) as a mediator was identified (Clements-Cortes, 2011). This was an intervention focused on music therapy. The results of this study indicated that examining life, signing songs, and creating musical gifts were at the heart of each participant's process. Grief, strength/hope, courage, but also gratitude and developmental growth were among the experiences of all participants. While all participants used their last weeks and days to live instead of waiting to die, they also invested this space in order to say goodbye to their loved one (often through their musical actions rather than verbally). Finally, love was the central feeling that had to be conveyed by all participants to help them complete their relationship.

#### Support Therapy

Similarly, only one support-oriented intervention was identified. Support for the couple promotes ”emotional discharge“ (Reny, 2020b). This is a means of re-circulating speech within the couple. What is more, through support and mediation work (between caregivers and couples), the intimacy of the subject and the couple can be heard and recognized by the caregiver. Finally, this is a space where “the couple they were before the critical illness” can be mourned. This can lead to a new functioning of the couple and a new way of being together.

#### Communication and Intimacy Promotion

Finally, three studies tested the effect of couple therapies whose objectives are common to most forms of couples therapy (e.g., to promote communication and intimacy) without being linked to a particular current (Mohr et al., 2003; McWilliams, 2004; Mowll et al., 2015). These studies showed that the interventional support promoted communication in men, lifted the veil on the feelings of each other, clarified the divergences within the couple favoring subsequently communication and changes in behavior toward the other (including preparation for the end of life) (Mowll et al., 2015). For the patient, these therapies tend to decrease distress with regard to death and increase the perception of the positive aspects of the relationship (Mohr et al., 2003). Among spouses, there is a decrease in worries about death (Mohr et al., 2003). With these couple interventions, psychological growth is observed in both the patient and his/her partner. Patients and spouses trust each other more and have confidence in their ability to continue to grow as a couple even if their time is limited (McWilliams, 2004). This type of intervention can also make it possible to value a communication that is already functional within the couple (Mowll et al., 2015).

Finally, several studies conclude by stipulating that participating in couple therapy during the illness makes it possible to create a link with the psychologist (McWilliams, 2004; Mowll et al., 2015; Reny, 2020b). In this sense, it is often accepted that couple interventions can help with the transition to bereavement follow-up once the loved one is gone (McWilliams, 2004; Mowll et al., 2015; Reny, 2020b).

By way of summary, we can say that the interventions offered to the couple confronted with advanced cancer improve marital functioning (cohesion, satisfaction, consensus, trustful in relationship, developmental growth, new functioning) (McWilliams, 2004; Benzein and Saveman, 2008; McLean and Hales, 2010; Clements-Cortes, 2011; McLean et al., 2013; Reny, 2020b), help to learn and find new strategies to manage daily life (Benzein and Saveman, 2008), to unburden themselves (Benzein and Saveman, 2008) and to reduce the loss of meaning (Lantz and Ahern, 1998). For patients, the intervention can be a space to say goodbye (Clements-Cortes, 2011). What's more, it improves the perception of patients (with regard to the relationship and the partners) (Mohr et al., 2003; McLean and Hales, 2010; McLean et al., 2013). Finally, it reduces distress (Mohr et al., 2003; Wagner et al., 2016). For spouses, the intervention has the effect of reducing anxiety, depression and worries about death (Mohr et al., 2003; Wagner et al., 2016) and increasing feelings of peace about the illness and perceptions of coping ability (Wagner et al., 2006).

## Discussion

The two objectives of this systematic review of the literature were (1) to explore scientific actual knowledge about the dyadic experience of the patient and his/her life partner when they are confronted with advanced cancer; (2)to highlight the impact of psychosocial interventions that are offered to the couples in the context of an advanced cancer.

Faced with often uncertain and ambiguous circumstances, cancer patients and their partners describe the individual and dyadic processes in which they have engaged as they approach the end of life (Gardner, 2008). Changes in the relationship were mainly focused on care, proximity/distance regulation, and modes of communication (Drabe et al., 2016). Communication appears to be an essential factor for both individual and dyadic adjustment (Cort et al., 2004; McLean and Jones, 2007; Drabe et al., 2016; Iwasaki et al., 2018; Mah et al., 2020; Reny, 2020a). Moreover, through better couple communication, attachment security supports preparation for death in cancer (Mah et al., 2020). Patient and spouse attachment styles contribute to spouse caregiving patterns (McLean and Jones, 2007; Braun et al., 2012). Indeed, the insecure attachment style and the resulting ”request-withdrawal“ and ”avoidance-chase“ couple interaction patterns are potential sources of distress (Braun et al., 2012). In addition, stress communication between partners and negative dyadic coping behaviors are correlated with high supportive care needs (Weißflog et al., 2017). Finally, dyadic adaptation has also been studied from the perspective of nutrition-related problems in advanced cancer: it is a dynamic and interactive process that relies on different adaptation pathways (Opsomer et al., 2019).

These results, particularly those related to communication, are consistent with the results found among the cancer population (Li and Loke, 2014). Our results suggest that for the population facing advanced cancer, psychotherapeutic interventions should be oriented toward a target population (e.g., young people, people with anxiety or insecure attachment, etc.) and focus on certain topics as applicable (e.g., communication, intimacy, adaptive behavior, problems specific to advanced cancer, etc.).

However, based on the critical analysis of the integrated studies, these results are based on limited evidence (low quality research plan and heterogeneous quality of study). Our results (and our avenues for reflection) should therefore be taken into consideration (as there are no others), but with a critical eye. They allow us to think about the relationship between the couple and advanced cancer, without asserting that a conclusion proposed in a study is a general truth. This is all the more true since there is a great diversity in the ways of apprehending the experience of the couple facing advanced cancer (relationship to food, communication, intimacy, temporality, etc.). This diversity, although very enriching, is confronted with the lack of existing studies, which means that there is little or no possibility of comparing the results of our studies (except for communication).

The interventions identified in our review focus on certain areas of interest identified above (e.g., communication, intimacy). All interventions identified have common general objectives: the promotion of communication and intimacy within the couple. Couple therapy, as a complement to end-of-life care, generates a relationship of trust between the couple and the therapist (which already appears to be a beneficial experience from the perspective of the dyad), gives couples the opportunity to express their concerns together, to identify differences in understanding, and to learn to find new strategies to manage daily life (Benzein and Saveman, 2008; Mowll et al., 2015; Reny, 2020b). It reduces the “meaning vacuum” and improves marital functioning. It also improves patient experiences (e.g., significant decrease in patients' distress at the thought of dying, perception of the threat) and that of partners (e.g., significant decrease in the frequency of partners' worries about the death of their partner, anxiety, depression, significant increase in peace with regard to illness, and perception of coping ability) (Lantz and Ahern, 1998; Mohr et al., 2003; McLean and Jones, 2007; Benzein and Saveman, 2008; McLean and Hales, 2010; McLean et al., 2013; Reny, 2020b). Finally, couple therapy has the potential to mitigate a catastrophic end of life and therefore a complicated marital bereavement, and it is also an easier transition to the accompaniment of the partner during the time of mourning, once the loved one is gone (McLean and Jones, 2007; McLean and Hales, 2010; Mowll et al., 2015; Reny, 2020b). We note that if the objectives remain the same, several models of interventions are proposed: existential, humanistic/systemic, psychodynamic and cognitive-behavioral. This is consistent with the 4 major models of psychotherapeutic interventions that are recognized as being able to be proposed in palliative care (Van Lander et al., 2015).

Based on the critical analysis of the integrated studies, these results can be said to be based on limited evidence (low quality research plan and heterogeneous quality of study). Our results must therefore be interpreted with caution. Normally, it cannot be used as the basis for a practical recommendation unless no other source of evidence is available; which is the case here. Consequently, we could develop clinical recommendations, but we will not do so and will only propose avenues for reflection. There is an obvious observation: using a robust methodology (and therefore obtaining evidence) in the context of the end of life is a real challenge. This raises the following question: should we focus on the level of evidence and exclude from review certain leads proposed by poor quality studies or should we lower our requirements regarding the level of evidence and adopt an inclusive approach? (We made the choice to reduce our level of requirements with regard to the field of study in which we are enrolled: that of the end of life).

Through these results, one can see that communication is an axis that has been widely studied by descriptive and interventional research. This is consistent with all models of dyadic adjustment that consider communication as a fundamental element (Manne and Badr, 2008; Fletcher et al., 2012). In a literature review that focuses on cancer, it was found that better communication between couples was linked to less distress and better marital adjustment (Li and Loke, 2014), which we find in our results on the couple facing advanced cancer. Communication could also be an element underlying the two other fundamental dimensions to the apprehension of the dyad: ”reciprocal influence“ and ”congruence“ (Fletcher et al., 2012; Li and Loke, 2014). These dimensions illustrating the concept of ”reciprocal influence“ (e.g., attachment style—-caregiving, nutrition-related problems, mutual optimism/positive approach) and ”congruence“ (e.g., almost total congruence on all the problems that the couple encounters in the face of advanced cancer, incongruence on beliefs and fears and subjective concerns related to death) are much less studied in an advanced cancer population than in the overall cancer population. In general, for the cancer population, concordance in dyads was linked to better individual outcomes and relationship satisfaction (Li and Loke, 2014). In the only study that evokes congruence within our population, this seems to be true: having divergent beliefs within the couple could interfere with mutual support (Gardner, 2008). These dimensions can very well be integrated into the systemic and transactional model of dyads (Untas et al., 2012). Integrating these elements into this model opens up the field of possibilities both in the clinic and in research.

Finally, the notion of temporality included in the model of Untas et al. (2012) is fundamental. While the temporality and trajectory of the disease are still largely underestimated in research on family care in oncology, we approach them essentially from a biological perspective based on pathological and radiological results (stage I-IV, early stage VS advanced stage). While this perspective is useful for defining groups for comparison in cross-sectional research, the evidence on disease stage in relation to the caregiving stress process is mixed (Fletcher et al., 2012). Future studies may show more definitive relationships between the stage of the disease and the stress process and researchers may also find that other ways to conceptualize the trajectory of cancer would be more fruitful. Indeed, we could conceptualize the trajectory of cancer in terms of phases that reflect the daily experiences of patients and family caregivers (”critical moments,“ ”nodal points in the trajectory of the disease,“ etc.) and thus consider periods of transition.

Despite the methodological robustness (linked to the Cochrane methodology) that guided this systematic exploration of the literature, some limitations should be highlighted. While we followed the recommendations of Cochrane, the selected studies include various risks of bias due to their design: selection bias, response bias, social desirability bias, absence of psychometric data on certain questionnaires used, small sample size, non-generalizable results, variables not considered, correlational effects and not causality, little diversity, high attrition rate, use of established tools and criteria that are not necessarily those recognized at the present time current. In addition, from one study to another, we observe significant differences (e.g., theoretical bases, methodologies and various protocols) which leads to difficulties in comparing our results.

## Conclusion

A relatively small number of articles were eligible for inclusion in this review, demonstrating the lack of evidence on the experiences of patients and life partners facing advanced cancer, but also the lack of evidence on the effectiveness of couple interventions. This observation limits the current clinical perspectives in terms of solid management of these dyads and conversely, it offers a multitude of research perspectives.

This literature review reveals that consideration (both psychological and physical) of the couple within the care appears fundamental. What is more, communication within the patient-life partner dyad is an essential axis of work. While many other variables appear important in the couple's dyadic adjustment to advanced cancer, there is very little evidence on the couple facing advanced cancer, though we know how severely the couple unit is impacted by cancer (Dankoski and Pais, 2007; Hagedoorn et al., 2008).

Descriptive and analytic studies are needed to advance research in this area. Indeed, longitudinal studies may, for example, be relevant to follow couples prospectively and assess the effect of the course of the disease on the couple (e.g., interactional patterns, emotional responses, dyadic adjustment strategies etc.). It could also provide elements of response relating to the apprehension of the trajectory of the disease by each partner of the dyad and from an interactional point of view. Also, qualitative protocols would allow a better understanding of the phenomena studied, in particular the representations that each partner has of the illness, the end of life, the post- death, etc. and the possible conflicts or difficulties resulting from a representational incongruence between them both. That is why, an openness to the concepts of ”reciprocal influence“ and ”congruence“ seems fundamental in order to open up our research perspectives in this field. Finally, experimental studies are needed (e.g., randomized controlled trials) to scientifically test the impact of specific dyadic therapies for couples facing advanced cancer and at the end of life. These research approaches would enable greater understanding of the complexity of the issues surrounding the dyadic experience of couples facing advanced cancer and allow effective psychosocial interventions to be proposed that are as close as possible to the realities of individual and dyadic experiences.

## Data Availability Statement

The original contributions presented in the study are included in the article/supplementary material, further inquiries can be directed to the corresponding author/s.

## Author Contributions

MH and BQ participated in the overall research and writing of the article. Both authors contributed to the article and approved the submitted version.

## Conflict of Interest

The authors declare that the research was conducted in the absence of any commercial or financial relationships that could be construed as a potential conflict of interest.

## Publisher's Note

All claims expressed in this article are solely those of the authors and do not necessarily represent those of their affiliated organizations, or those of the publisher, the editors and the reviewers. Any product that may be evaluated in this article, or claim that may be made by its manufacturer, is not guaranteed or endorsed by the publisher.

## Footnotes

^1^Communication:“a transactional process in which individuals create, share, and regulate meaning.” Reciprocal influence: “the effect the two members of a dyad have on each other.” Caregiver patient congruence: “the concept of congruence synthesizes individual data into a dyad variable, related to agreement, concordance, and their opposite, disparity” (Fletcher et al., 2012).

^2^Caregiving proximity – comfort with physical closeness during support provision; Sensitive caregiving – attuned responsiveness to partner's signals and needs; Controlling caregiving – a domineering style of caregiving, which lacks sufficient respect for partner's own problem-solving and decision making processes; Compulsive caregiving – a tendency to be over involved and over-protective, often being overwhelmed by extreme identification with partner's problems (Braun et al., 2012; McLean and Jones, 2007).

## Appendix

For each database we used the thesaurus when available (structured directory of terms for content analysis and document classification). In the table below, you will find the terms that are covered by the thesaurus keywords. The “+” equals other integrated terms not expanded.

**Table:**

	**“Couple”**	**“Palliative care”**
PubMed	Family Characteristics Couples Therapy	Palliative care Hospice and Palliative Care Nursing Palliative Medicine
PsycArticle	Interpersonal relationships Same sex Couples cohabitation + Dating violence Dyads Family + Online dating Romance Significant others Social dating Spouses +	Health care services Assisted suicide Euthanasia Advance directives Death and dying + Health care delivery + Hospice Life sustaining treatment + Long term care Pain management Symptoms based treatment Terminally ill patients End of life care
PsycInfo	Interpersonal relationship Same sex couples Cohabitation Dating violence Dyads Family + Online dating Romance Significant others Social dating Spouses +	Health care services Assisted suicide Euthanasia Advance directives Death and dying + Health care delivery + Hospice Life sustaining treatment Long term care Pain management Symptoms based treatment Terminally ill patients End of life care
Behavorial Sciences Collection	Interpersonal relations Academic couples African american couples Architect couples Criminal couples Gay couples Infidelity (couples) Interethnic couples Intergenerational couples Interracial couples Lgbtq+ couples Life partners Married people Older couples Royal couples Unmarried couples Conjoint therapy Couples in art Couples in literature Couples in motion pictures Couples on television Crimes against couples Dating (social customs) Dyadic communication Dyads Primary groups (social groups) Relationship status Sexual partners Heterosexual couples	Therapeutics Brompton cocktail Hospice care Palliative medicine Palliation (medical care) Palliative treatment
